# Bimatoprost Effects on Aqueous Humor Dynamics in Monkeys

**DOI:** 10.1155/2010/926192

**Published:** 2010-05-23

**Authors:** David F. Woodward, Achim H.-P. Krauss, Siv F. E. Nilsson

**Affiliations:** ^1^Department of Biological Sciences, Allergan, Inc., Irvine, CA 92612, USA; ^2^Ophthiris, GlaxoSmithKline, King of Prussia, PA, USA; ^3^Division of Drug Research, Department of Medical and Health Sciences, Faculty of Health Sciences, University of Linköping, Linköping, Sweden

## Abstract

The effects of bimatoprost on aqueous humor dynamics were quantified in monkey eyes. Uveoscleral outflow was measured by the anterior chamber perfusion method, using FITC-dextran. Total outflow facility was determined by the two-level constant pressure method. Aqueous flow was measured with a scanning ocular fluorophotometer. Uveoscleral outflow was 0.96 ± 0.19 *μ*L min^−1^ in vehicle-treated eyes and 1.37 ± 0.27 *μ*L min^−1^ (*n* = 6; *P* < .05) in eyes that received bimatoprost 0.01% b.i.d. × 5 days. Bimatoprost had no effect on total outflow facility, which was 0.42 ± 0.05 *μ*L min^−1^ at baseline and 0.42 ± 0.04 *μ*L min^−1^ after bimatoprost treatment. Bimatoprost had no significant effect on aqueous humor flow. This study demonstrates that bimatoprost increases uveoscleral outflow but not total outflow facility or aqueous humor flow, indicating that it lowers intraocular pressure in ocular normotensive monkeys by a mechanism that exclusively involves uveoscleral outflow.

## 1. Introduction

Bimatoprost (Lumigan) is a highly efficacious ocular hypotensive agent, [1–5] with a unique pharmacology [1]. It mimics the activity of the prostaglandin ethanolamides (prostamides), which are formed from the endocannabinoid anandamide by cyclo-oxygenase-2 (COX-2) [6–9]. Several studies have demonstrated that the pharmacology of the prostamides and bimatoprost is distinct from that of the prostaglandins [1, 10–16] and the endocannabinoids [17]. The differentiated pharmacology of bimatoprost is also manifest in the clinical setting where it lowers intraocular pressure (IOP) in open-angle glaucoma and ocular hypertensive patients who are nonresponders to the FP receptor agonist prodrug latanoprost [18–20]. 

Measurement of uveoscleral outflow in patients, of necessity, is derived indirectly. It is calculated from measurements of aqueous humor flow, outflow facility, and an assumed typical value for episcleral venous pressure. Direct measurement of uveoscleral outflow requires enucleation of the eyes and measurement of a radiolabeled or fluorescent marker in anterior segment tissues. The purpose of this study was to provide a direct measurement of uveoscleral outflow in primates, of a nonhuman variety. Total outflow facility was also measured directly by the two-level constant pressure method. For completion, aqueous humor flow was measured fluorphotometrically.

## 2. Materials and Methods

Ocular normotensive cynomolgus monkeys (Macaca fasciculate) of both sexes and weighing between 2.2 and 4.0 kg were used for these studies. All experiments were performed according to the ARVO statement for the Use of Animals in Ophthalmic and Vision Research. All animals were determined as suitable for study according to prior slit-lamp biomicroscopy. For measurement of total outflow facility and uveoscleral outflow, one eye received 25 *μ*L of 0.01% bimatoprost at ~6 A.M. and again at ~2 P.M. to provide B.I.D. dosing. The contralateral eye received vehicle (5 mM TRIS-HCl/0.1% polysorbate 80) in an identical manner. Each animal received a total of 9 doses in each eye over a four and one-half day period. Between 5 and 7 hours after the final dose, the animals were anesthetized by intramuscular injection of 0.3 mL/kg of a mixture of ketamine (25 mg/mL) and xylazine (10 mg/mL) to provide deep anesthesia and an absence of eye movements. Anesthetic was supplemented during the course of the experiment at a dose of 0.1 mL/kg of the mixture when the level of anesthesia was reduced to the point where first signs of slow eye movements and blinking became apparent. Supplemental doses of the anesthetic mixture were typically given 45–60 minutes after the initial dose or even earlier in some instances. Vital signs, heart rate and body temperature, were continuously monitored. 

Outflow facility studies were performed as follows. After a stable and deep level of anesthesia had been achieved, both eyes were cannulated through the cornea with one 25-gauge stainless steel needle by using a needle gun. The IOP was determined with a calibrated pressure transducer system. Outflow facility was then determined with a 2-level constant pressure infusion method described by Bárány [21]. In brief, the anterior chamber was connected in series to a pressure transducer and a fluid reservoir containing a modified mock artificial aqueous humor solution [22]. The pressure in the eye was altered at 10-minute intervals between two different pressure levels approximately 2.5 (*p*_1_) and 10 mm Hg (*p*_2_) above the “real” IOP level. “Real” IOP in this context describes the IOP that would be present in the eye if the pressure was not artificially maintained with the reservoir. For each 10-minute measurement period at *p*_1_ or *p*_2_, the flow rate of fluid from the reservoir was determined as *F*_1_ or *F*_2_, respectively. Flow rates were calculated as *F* = Δ*W*/*t* with Δ*W* representing the weight difference of the reservoir between the beginning and end of the measurement period and *t* representing the duration of the period. Total outflow facility *C* could then be calculated with the equation

(1) $$C=\frac{F_{2}-F_{1}}{\left(p_{2}-p_{1}\right)}.$$

Total outflow facility values in each eye were averaged over 5 measurement periods. The outflow facility studies were done twice in the same animals approximately 4 months apart. Total outflow facility was first measured in naïve, untreated animals (to obtain a “baseline”) and four months later after 5 days of bimatoprost unilateral treatment (as described above).

To determine the effect of bimatoprost on uveoscleral outflow, we used the anterior chamber perfusion method originally developed by Bill [23] and then modified by Toris et al. [24] using a fluorescein-tagged tracer instead of radiolabeled albumin. Each eye was cannulated with three 25-gauge needles, two of which were connected to 5 mL gastight syringes mounted in a Harvard reciprocal syringe pump, the third was connected to an open fluid reservoir with an inline pressure transducer to record IOP. The Harvard reciprocal pump allowed the perfusion of the anterior chamber at a predetermined flow rate without affecting IOP by virtue of the perfusion process. Both eyes were perfused for 30 minutes with a mock aqueous humor solution [22] containing 0.7% (1 × 10^−4^ M) FITC-dextran 70,000 as a tracer. To provide for a quick exchange of the anterior chamber content with the tracer, the eyes were perfused for the first 5 minutes at a rate of 0.2 mL/min and then during the remaining 25 minutes at a rate of 0.05 mL/min. The perfusates from those two perfusion periods were collected separately as primary and secondary perfusate, respectively. The IOP in both eyes was set during the anterior chamber perfusion at approximately 12 mm Hg via the open reservoir. At the conclusion of the 30-minute perfusion period the animal was euthanized with an overdose of Eutha-6 CII (2 mL). Both eyes were then perfused for approximately 10 minutes with mock aqueous humor solution, without tracer, to wash the tracer out of the anterior chamber. The eyes were then enucleated and immediately dissected into the following tissues: anterior sclera, posterior sclera (posterior to ora serrata), extraocular tissues, ciliary body, choroid, retina, vitreous humor, and other fluids including wash fluid. Cornea, lens, and iris were excluded from the measurements because these tissues are not thought to contribute to uveoscleral outflow. 

The amount of tracer was determined in each ocular tissue separately. For that purpose, each tissue was homogenized in Dulbecco's phosphate-buffered saline (D-PBS). The FITC-dextran concentration in tissue homogenates was determined with a Perkin-Elmer LS-50B spectrometer using excitation and emission wavelengths of 493 and 515 nm, respectively, and 5.0 nm excitation and emission slits. Total uveoscleral outflow (*F*_*u*_) was calculated with equation

(2) $$F_{u}=\Sigma \left(\text{tissue-FITC}\right)\times {\left[\text{perfusate-FITC}\right]}^{-1}\times {\text{time}}^{-1},$$

where Σ(tissue-FITC) represents the total amount of FITC-dextran present in all ocular tissues, [perfusate-FITC] represents the actual FITC-dextran concentration in the anterior chamber perfusate (which was collected as secondary perfusate with the receiving syringe during the 25-minute perfusion period), and time represents the duration of the perfusion (i.e., 30 minutes). 

Statistical analysis was performed with Students's *t*-test for paired observations using the software package StatView Version 4.51 for Windows. Differences were assumed to be statistically significant for *P* < .05.

Aqueous flow was measured fluorophotometrically with a Fluorotron instrument (OcuMetrics, Mountain View, CA). The animals were briefly restrained during the procedure in custom-designed chairs. Background autofluorescence scans were taken on the afternoon prior to the fluorophotometry experiment. The experiment was commenced by giving one drop of 0.5% proparacaine (Ophthetic, Allergan) to each eye. Five minutes later a drop of proparacaine followed by 2 *μ*L Na fluorescein (2%) was given. This proparacaine/fluorescein dosing procedure was repeated four times at five minutes intervals. This procedure “loaded” the eye with fluorescein and provided steady-state conditions for the experiment on the following day. Fluorphotometric measurements were performed 7 times, one hour apart, on the following day. Each eye was scanned at least twice per reading. Ketamine (1.4 mg/kg) was injected intramuscularly 5 minutes before each scan. 

In order to calculate aqueous humor flow, values for anterior chamber depth and corneal thickness and curvature were obtained. Anterior chamber depth and corneal thickness were determined by pachymetry using a Haag-Streit slit lamp. Corneal curvature was determined using a keratometer. These values allowed anterior chamber volume to be calculated. Aqueous humor flow was obtained by software written for the IBM or Macintosh by M.A. Croft (University of Wisconsin) according to equations developed by Yablonski [25]. 

For aqueous humor flow studies with bimatoprost, (0.1%) the drug was administered twice on the day before the flow experiment with a 6-hour interval between doses and again on the morning of the day of the flow experiment in a different series of experiments. Timolol, employed as a reference drug, was administered once on the day of the flow experiment in a separate series of the experiments involving unilateral administration of timolol, the contralateral eye receiving the vehicle as a control.

## 3. Results and Discussion

### 3.1. Aqueous Humor Dynamics

The effects of bimatoprost (0.01%) on total outflow facility and uveoscleral outflow in monkeys were determined approximately 6 hours after the 9th dose of treatment on day 5 of a 5-day BID dosing regimen.

In the untreated, naïve study animals total outflow facility was similar in the right and left eye and is shown as “Baseline” values in Table 1. No significant effect on total outflow facility was observed after unilateral treatment with bimatoprost (0.01%) as shown in Table 1. The uveoscleral outflow in the eyes treated with bimatoprost (0.01%) was 1.37 ± 0.27 *μ*L/min compared to 0.96 ± 0.20 *μ*L/min in the contralateral control eyes as shown in Table 2. This represents a 42% increase. 

The aqueous humor flow rates comparing the effects of 3 × 0.1% bimatoprost and a single dose of timolol are provided in Table 3. Although timolol, employed as a positive control, effectively suppressed aqueous humor inflow by about 42%, bimatoprost exerted no meaningful effect.

## 4. Discussion

Studies in normal human volunteers and in patients with ocular hypertension or glaucoma have shown that bimatoprost increases both pressure-sensitive and pressure-insensitive aqueous humor outflow. The latter is presumed to reflect uveoscleral outflow [26, 27]. These clinical determinations of uveoscleral outflow were calculated from measurements of aqueous humor flow and trabecular outflow using the Goldmann equation, since direct measurement of uveoscleral outflow requires enucleation and dissection of ocular tissues. Thus, one objective of profiling the effects of bimatoprost on monkey aqueous humor dynamics was to provide direct assessment of uveoscleral outflow. Bimatoprost, in a dosing regimen that produces profound and long-lasting decreases in intraocular pressure in normal monkeys [1], was found to produce a significant increase in uveoscleral outflow. This direct physiological measurement of uveoscleral outflow, together with morphological studies [28], is consistent with the notion that bimatoprost does indeed increase uveoscleral outflow. 

Bimatoprost exerted no meaningful effect on total outflow facility or aqueous humor flow in cynomolgus monkeys and it may be presumed that increased uveoscleral outflow provides a singular explanation for its ocular hypotensive effects in this species. The dosing regimens used in this study are experimental and differ from that currently used clinically, which is 0.03% once daily. It is difficult to offer a straightforward explanation for the inability of total outflow facility results obtained in cynomolgus monkeys to predict marked increases in tonographic outflow in humans. They do not necessarily equate. It is possible that the anesthesia, which is obligatory for direct measurement of total outflow facility in monkeys, may have blunted trabecular outflow by affecting the normal function of trabecular meshwork cells and endothelial cells of Schlemm's canal. This possible explanation should, however, be viewed with some caution. Sex and age do not provide an obvious explanation; the ages of the normal volunteers [26] and the monkeys used in these studies were comparable taking into account the life-span of the two species. It is most unlikely that the dosing regimen used in the monkey studies (0.01% bimatoprost, twice daily for 5 days) fails to produce changes in outflow facility that can be observed in the clinical setting with a dosing regimen of 0.03% once daily. Bimatoprost (0.01%) given twice daily produces profound effects on monkey IOP (1). Species difference [29] with respect to pharmacological control of aqueous humor dynamics may provide a more plausible explanation, despite the fact that monkeys and man are phylogenetically very close. 

The monkey is preferred for ocular studies by virtue of possessing an ocular anatomy and physiology that appears very similar to that observed in humans, the only obvious difference being the size of the globe. There is, however, some evidence to suggest that the monkey does not necessarily always mimic the human eye in all respects. The *α*_2_ adrenoceptor agonist brimondine provides a further example. In monkeys there is a centrally mediated bilateral response to brimondine [29], that is not apparent in human subjects [30]. The 11,15-dipivaloyl ester of PGF_2*α*_ exhibited acceptable efficacy in monkeys [31] but was devoid of any ocular effects in humans (unpublished data). Prostanoid FP receptor agonists such as travoprost and latanoprost have also been reported to exert no meaningful effect on trabecular outflow in monkeys [32, 33]. Effects on presumed trabecular outflow facility may, however, occur in human subjects but there is conflicting data. Using Schiotz tonography latanoprost exerted an apparent effect on pressure-sensitive outflow [34] whereas other studies using alternative but seemingly valid techniques showed no effect on conventional outflow facility [35]. It is possible that the increase in conventional outflow observed in human eyes does not really exist [35]; this is the case in primate eyes. As a model species for predicting the ocular effects in humans, the cynomolgus monkey should perhaps be regarded as very useful but not infallible.

## 5. Conclusions

The data confirm that bimatoprost exerts an effect on uveoscleral outflow by direct measurement in cynomolgus monkeys. Bimatoprost effects on conventional outflow observed in humans [26, 27] were not detected in monkeys. In cynomolgus monkeys, bimatoprost appears to lower IOP by exclusively increasing uveoscleral outflow. 

## Figures and Tables

**Table 1:** Effect of bimatoprost (0.01%) administered twice daily for 5 days on total outflow facility in (*μ*L min^−1^ mm Hg^−1^) cynomolgus monkeys, *n* = 6 (Mean ± SEM).

Total outflow facility (*μ*L min^−1^ mm Hg^−1^)		
	Vehicle (OS)	Treated (OD)

Baseline	0.46 ± 0.03	0.42 ± 0.05
Treatment	0.37 ± 0.02	0.42 ± 0.04

**Table 2:** Effect of bimatoprost (0.01%) administered twice daily for 5 days on uveoscleral outflow (*μ*L min^−1^) in cynomolgus monkeys *P < .05, *n* = 6 (Mean ± SEM).

Uveoscleral outflow (*μ*L min^−1^)	
Bimatoprost	1.37* ± 0.27
Vehicle	0.96 ± 0.20

**Table 3:** Comparison of the effect of timolol (0.5%) and bimatoprost (0.1%) on aqueous humor flow (*μ*L/min^−1^) in cynomolgus monkeys, *P < .05, *n* = 6 (Mean ± SEM).

Aqueous humor flow (*μ*L min^−1^)		
	Timolol (0.5%)	Bimatoprost (0.1%)

Vehicle	1.64 ± 0.14	1.41 ± 0.07
Drug	0.91 ± 0.09	1.497 ± 0.07
